# PReS-FINAL-2319: PED-BD cohort 2013: expert consensus classification gives higher sensitivity than the international study group criteria to define Behcet's disease in children

**DOI:** 10.1186/1546-0096-11-S2-P309

**Published:** 2013-12-05

**Authors:** I Koné-Paut, M Darce Bello, F Shahram, S Ozen, R Cimaz, H Michael, M Gattorno, B Chkirate, K Bouayed, L Cantarini, J Anton, I Tugal-Tuktun, J Kummerle-Deschner, A Faye, S Benamour, TA Tran, A Arnoux, PED-BD

**Affiliations:** 1 Pediatric Rheumatology, Bicêtre University Hospital, Le Kremlin Bicêtre, France; 2 Rheumatology Research, Shariati Hospital, Tehran, Iran, Islamic Republic of; 3 Pediatric Nephrology, Hacettepe University, Ankara, Turkey; 4 Pediatric Rheumatology, Meyer Hospital, Florence, Italy; 5 Pediatric Rheumatology, Vaudois University Hospital, Lausanne, Switzerland; 6 Pediatric Rheumatology, G. Gaslini, Genoa, Italy; 7 Pediatrics, Rabat University Hospital, Rabat, Morocco; 8 Pediatrics, Casablanca University hospital, Casablanca, Morocco; 9 Medicine and Immunological Sciences, University of Siena, Siena, Italy; 10 Pediatric Rheumatology, Sant Joan de Déu Hospital, Esplugues de Llobregat, Spain; 11 Ophtalmology, Istanbul University, Istanbul, Turkey; 12 Pediatric Rheumatology, University of Tuebingen, Tuebingen, Germany; 13 Pediatrics, R Debré University Hospital, Paris, France; 14 Internal Medicine, Casablanca University Hospital, Casablanca, Morocco; 15 Clinical Research, Bicêtre University Hospital, Le Kremlin Bicêtre, France

## Introduction

BD is rarely encountered in children where the disease is very difficult to recognize. The outcome ofpatients with few symptoms is currently unknown

## Objectives

To define the outcome of paediatric patients with at least two symptoms of BD, and to obtain an appropriate definition of BD in patients <16y.

## Methods

An international expert committee has defined the criteria of inclusion. New patients or patients followed for a maximum of 3 years, who presented at least 2 symptoms of BD (among a list), and gave their informed consent were included, reviewed yearly.

## Results

228 patients were included since 2008, (SR: 1), from 22 centres of 13 countries, median age of 12.5y. Median age at first symptom was 7.2y. Family history of BD was present in 22% and consanguinity in 4.5%. Median disease duration at inclusion was 4.7y and from the first symptom to last visit was 7.5y. Inclusion criteria plus oral aphtosis (mandatory) were (%): genital aphtosis 50, necrotic folliculitis 31, uveitis 28, familial history 22, pathergy positive 19, erythema nodosum 15, vascular 10 and retinal vasculitis 7. Mean number of symptoms: 1 plus family history 41%, 2 (33%), more than 3 (26%). Patient had a median of 1.3 follow-up visit (0-4). 220 patients had a first visit, 138 patients had a 1-y visit (mean BD duration: 5.8y). 81 patients had 2-y (6.4y), 44 a 3-y (7.3y) and 18 a 4-y visit (7.5y). The symptoms along the study were (%): dermatological 67, genital aphtosis 52, articular 48, fever 47, gastrointestinal 39, ocular 36, neurological 35, pathergy 17, vascular 12, urological 2. HLAB51 was present in 47%. Male patients had significantly more ocular and vascular signs, female had more genital aphtosis. Between 1st-4th visit: 57% had no new symptom, 24% had 1, 11% had 2 and 10% had more than 3. The expert committee has examined 199 files at a median disease duration of 6,1y, and classified 121 patients as definite, 18 as probable and 3 as not BD. 57 charts were reviewed but did not reached consensus. 46 files have been reviewed more than once.. Among our patients classified as definite: 121/142 (85%); 79/121 (65%) fulfilled the ISG International criteria. International criteria and expert classification showed significant differences. Although good concordance (Kappa c = 0.72). Having 2 or more symptoms was significantly associated with classification as definite BD (p = 0.0005).

## Conclusion

The expert committee has classified the majority of patients in the BD group although they did not fulfil the international BD classification criteria (for adults).

## Disclosure of interest

None declared.

